# The independent adverse prognostic significance of 1q21 gain/amplification in newly diagnosed multiple myeloma patients

**DOI:** 10.3389/fonc.2022.938392

**Published:** 2022-10-07

**Authors:** Hongying You, Song Jin, Chunxiao Wu, Qingqing Wang, Shuang Yan, Weiqin Yao, Xiaolan Shi, Jingjing Shang, Lingzhi Yan, Ying Yao, Jing Wang, Panfeng Wang, Jinlan Pan, Depei Wu, Chengcheng Fu

**Affiliations:** ^1^ National Clinical Research Center for Hematologic Diseases, Jiangsu Institute of Hematology, The First Affiliated Hospital of Soochow University, Suzhou, China; ^2^ Key Laboratory of Thrombosis and Hemostasis of Ministry of Health, The First Affiliated Hospital of Soochow University, Suzhou, China; ^3^ Hematology Department, Suzhou Hongci Hematology Hospital, Suzhou, China

**Keywords:** multiple myeloma, 1q21 gain/amplification, fluorescence *in situ* hybridization (FISH), CytoScan, prognostic analysis

## Abstract

**Objective:**

1q21 gain/amplification (1q21+) is a common abnormal karyotype in multiple myeloma, and its proportion in Chinese patients is much higher. If 1q21+ is included as one of the poor prognostic factors, it will greatly increase the proportion of high-risk patients in newly diagnosed multiple myelome (NDMM) patients. Therefore, the poor prognostic significance of 1q21+ is still controversial. This study mainly analyzed the clinical characteristics, treatment response and prognostic significance of 1q21+ in NDMM patients.

**Methods:**

248 NDMM patients admitted in The First Affiliated Hospital of Soochow University from September 01, 2018 to August 31, 2021 of a VRD registration study, were retrospectively analyzed. 135 cases (54.4%) had 1q21+ by CD38-sorted fluorescence *in situ* hybridization (FISH). The clinical characteristics, treatment response and prognosis of the general population and subgroups were analyzed, among which 153 patients were compared for the involved genes by CytoScan.

**Results:**

Compared with negative patients, 1q21+ patients were more likely to have anemia, hypoalbuminemia, renal insufficiency, high lactate dehydrogenase and high proportion of R-ISS-III stage. The patients with 1q21+ involving CKS1B detected by Cytoscan had a higher proportion of complex karyotypes and abnormal CNVs, and all at middle-risk or high-risk groups defined by Prognostic Index. Multivariate analysis showed that 1q21+ was an independent adverse prognostic factor (PFS HR=2.358, 95%CI 1.286-4.324, P=0.006; OS HR=2.598, 95%CI 1.050-6.425, P=0.039). 1q21+ subgroup had an inferior outcome (PFS P=0.0133, OS P=0.0293). Furthermore 1q21 amplification had a shorter PFS than 1q21 gain (24 months vs not reached, P=0.0403), but the OS difference was not clinically significant. The proportion of 1q had no effects on prognosis. In addition, 1q21+ in main clone rather than subclone was an adverse factor affecting the prognosis (PFS P=0.0172, OS P=0.1260). Autologous stem cell transplantation can effectively improve the survival of 1q21+ patients (P<0.05).

**Conclusion:**

Patients with 1q21+ have clinically significant end-stage organ damage and higher tumor burden, more likely to combine 13q14-, t(4;14), 1p32- and other cytogenetic abnormalities. 1q21+ is an independent high-risk cytogenetic factor for poor prognosis in NDMM patients, of which 4 or more copy numbers and main clone position significantly associated with prognosis results.

## Background

Multiple myeloma (MM) is a hematological malignancy with proliferation of monoclonal plasma cells which has complex genetics and prognostic heterogeneity. The survival varies from months to years. It continues to evolve due to the clonal evolution of tumors, the high instability of the genome, and the clonal heterogeneity (1), which is still incurable to date. The occurrence and accumulation of different genetic abnormalities contributes to the heterogeneous clinical progress of the disease. It involves a variety of primary genetic abnormalities, including t (4; 14), t (11; 14), t (14,16) and hyperdiploid karyotypes, as well as secondary genetic abnormalities, including chromosome copy number abnormalities (1q21+, 1p-, 13q-, 17p-, etc) and secondary translocations like MYC (2).

As the most common secondary genetic event, 1q21+ occurs in about 30-40% of patients at the time of initial diagnosis (3). The proportion is especially higher in Chinese population, accounting for 59.7% (96/161) by a domestic study in 2022 (4), it is consistent with a plasma cell disease description by Mao (47.4%, 376/789) (5) and a cytogenetic abnormalities epidemiology study of multiple centers by Yuan (46.1%, 468/1015) (6), at about 40-60%. Despite its frequency, considerable debate remains regarding the prognostic impact of 1q21 in MM. According to a recent meta-analysis of 2,596 trial patients (7), 1q21+ were both associated with shorter progression free survival (PFS) and overall survival (OS), as well as in the study of Yang et al (8), 1q21+ also had a statistical significantly difference in PFS and OS. However Li (9) had a further analysis revealed that in the absence of other high-risk factors, the differences in PFS (52.0 vs. 52.8 months, p =0.810) and OS (not reached vs. not reached, p=0.833) between patients with or without 1q21+ were not statistically significant. In contrast to factors like t(4;14), t (14,16) and del(17p), 1q21 status is not included among the high-risk markers listed by the International Myeloma Working Group R-ISS, and neither listed as a high-risk factor in Chinese guidelines, it was first emphasized adversely until mSMART3.0 (2018) (10). So, our center has begun to take 1q21 seriously as a heterogeneity problem that deserves to be studied and treated.

In the past two decades, chromosome karyotyping firstly analyzed the number or structure of chromosomes, but the result was limited by terminally differentiated MM cell numbers and low proliferation rate. By now, FISH is the most used detection method that uses special fluorescein-labeled DNA probes to detect the percentage of abnormal cells (1). Due to the deepened understanding of cytogenetics, limited probes cannot cover a variety of genetic abnormalities, so CytoScan is gradually promoted abroad, firstly as a new technology focusing on genetic disease diagnosis and prenatal diagnosis (11), and it has not been widely used in China. The technology analyzes DNA sequence polymorphisms caused by variation at the nucleotide level of the genome, including copy number changes at the genome-wide level. High-resolution detection of each chromosome can detect small abnormalities that cannot be identified by present technology analysis (12), promoting the complement and correction of FISH and CytoScan. This study will combine the two techniques to analyze the clinical characteristics, response and prognostic significance of NDMM patients with 1q21 gain/amplification.

## Methods

### Patients

This study included 248 NDMM patients who were admitted in The First Affiliated Hospital of Soochow University from September 1, 2018 to August 31, 2021. All patients were enrolled in a registered clinical trial and treated with VRD in combination with autologous stem cell transplantation or VRD treatment for 8 cycles. This trial belongs to Project Beyond Multiple Myeloma Database (PBMMD) which is a collaborative project in China to collect Real World Evidence (RWE) on the management and outcome of patients with Multiple Myeloma in China. This study was reviewed and approved by the Ethics Committee of the First Affiliated Hospital of Soochow University and informed consent of the patients. The patients first underwent a 4-cycle standard VRD induction treatment, which was subcutaneous injection of bortezomib 1.3 mg/m^2^ on days 1, 4, 8, and 11, and intravenous infusion of dexamethasone 20 mg/d on days 1, 2, 4, 5, 8, 9, 11, 12, and lenalidomide 25 mg/d from 1 to 21 days, 28 days as a treatment cycle. The efficacy was evaluated after 4 cycles of treatment, and then they were divided into a transplantation group (VRD combined with autologous stem cell transplantation) and a non-transplantation group (VRD continued for 8 cycles of treatment), and received maintenance therapy.

The diagnosis, staging and treatment response evaluation of MM refer to the 2017 revised version of Chinese guidelines for the diagnosis and treatment of multiple myeloma (13). Fluorescence *in situ* hybridization (FISH) results of patients before treatment indicated that positive t(4;14), t(14;16) or del(17p) were defined as cytogenetic high-risk, and the rest were standard-risk. Standard-risk patients were treated with lenalidomide alone for more than 2 years, and high-risk patients or R-ISS-III patients were treated with lenalidomide combined with proteasome inhibitors maintenance for more than 2 years. In this study, mSMART3.0 (version 2018) and CytoScan Prognostic Index (PI) were used to further analyze the prognostic significance of karyotype high-risk stratification criteria.

### Fluorescence *in situ* hybridization (FISH)

Cells were sorted by CD138 magnetic bead antibody, and all special fluorescein-labeled DNA probes, including 1q21 gain/amplification, 13q14 deletion, Rb1 deletion, IgH rearrangement, and 17p deletion, among which if IgH rearrangement was positive, then added t (4; 14), t (11;14), and t (14;16) detection. Through the steps of sorting, fixation, aging, dehydration, mounting, hybridization, counterstaining, etc., fluorescence microscope is used to observe the fluorescence hybridization signal of interphase cells under the excitation of different filters. At least 200 interphase cells in each case were analyzed and then calculate the percentage of cells showing abnormal signal. Abnormal threshold = mean + 3*standard deviation, the range above the threshold is positive, and the range below the threshold is negative.

### CytoScan

The CD138-sorted cell samples from 153 patients were obtained from the Multiple Myeloma Specialized Disease Bank of the National Center for Clinical Medical Research of Hematological Diseases, and the Blood Disease Sub-Bank of the Jiangsu Provincial Major Disease Biological Resource Sample Bank. A 750K DNA CytoScan (Affymetrix) was used to hybridize the sequence to be detected to achieve typing. According to the complementary base pairing, the sequence with a single mismatched base could not hybridize with the probe. Sites were detected by the difference in elution between hybrid molecules so called fluorescence intensity. Chromosome Analysis Suite (CHAS) software was used to analyze DNA sequence polymorphisms caused by variation at the nucleotide level of the genome, including copy number variation (CNV) at the genome-wide level, such as gains, deletions and their chimerism. It can be tested when the proportion of abnormal cells >10% of all tested cells, and the detection sensitivity and specificity can be >99%.

### Follow-up

All 248 patients were included in the final analysis, and the follow-up period was from the date of diagnosis to February 28, 2022. Follow-up was conducted through inpatient medical records, outpatient medical records and telephone calls. Cases who died during the follow-up period were confirmed by medical records and/or the family through calls. Survival measures were by OS and PFS: Overall survival (OS) was defined as the time from the date of diagnosis to the time of last follow-up or the date of death. Progression-free survival (PFS) was defined as the time from diagnosis to disease progression or death.

### Statistical analysis

Statistical analysis was performed using SPSS 25.0 software. Means were compared by independent samples t test and two-sided ANOVA test; rates were compared using χ^2^ test; Kaplan-Meier was used for overall survival; Log-rank test was used to estimate the survival difference of individual risk factors; Cox regression analysis was applied to univariate and multivariate regression scores. P<0.05 was statistically significant.

## Results

### Patient and baseline disease characteristics

Clinical and biological characteristics of 248 NDMM patients in this study are shown in **Table 1**. Compared with patients without 1q21+, positive patients were more likely to have anemia (74.8%, P=0.019), hypoalbuminemia (65.2%), renal insufficiency (23%), and high lactate dehydrogenase (19.2%) etc. The proportion of R-ISS-III in 1q21+ patients was significantly higher than that in patients without it (P=0.021).

**Table 1:** Clinical and biological characteristics of total patients and 1q21+ patients.

Characteristics	Total (n=248)	1q21+ (n=135)	1q21- (n=113)	P value
Age [years, range]	60 (31-82)	63 (39-82)	60 (31-83)	0.751
Gender [male (%)]	135 (54.4)	76 (56.3)	59 (52.2)	0.524
Type [n (%)]				0.380
IgA	50 (20.2)	30 (22.2)	20 (17.7)	
IgG	100 (40.3)	52 (38.5)	48 (42.5)	
IgD	8 (3.2)	4 (3.0)	4 (3.5)	
Light chain	50 (20.2)	23 (17.0)	27 (23.9)	
Other	40 (16.1)	26 (19.3)	14 (12.4)	
DS stage [n (%)]				0.732
I、II	9 (3.6)	6 (4.4)	3 (2.7)	
IIIA	169 (68.1)	92 (68.1)	77 (68.1)	
IIIB	70 (28.3)	37 (27.4)	33 (29.2)	
ISS stage [n (%)]				0.068
I	37 (14.9)	14 (10.4)	23 (16.8)	
II	115 (46.3)	65 (48.1)	50 (44.2)	
III	96 (38.7)	56 (41.5)	40 (35.4)	
R-ISS stage [n (%)]				0.021
I	30 (12.1)	11 (8.1)	19 (16.8)	
II	168 (47.8)	92 (68.1)	76 (67.3)	
III	50 (20.1)	32 (23.7)	18 (15.9)	
Hemoglobin [<100g/L, n (%)]	169 (68.1)	101 (74.8)	68 (60.2)	0.019
Albumin[≤ 35g/L, n (%)]	154 (62.1)	88 (65.2)	66 (58.4)	0.294
Serum creatinine [>177umol/L, n (%)]	48 (19.4)	31 (23.0)	17 (15.0)	0.116
Serum calcium [>2.75mmol/L,n (%)]	11 (4.4)	4 (3.0)	7 (6.2)	0.235
β2-microglobulin [>3.5mg/L, n (%)]	141 (56.8)	78 (57.8)	63 (55.8)	0.748
24hurinary protein [>5g/24h, n (%)]	22(22/187, 11.8)	9 (9/108, 8.3)	13 (13/79, 16.4)	0.088
Lactate dehydrogenase [>250U/L, n(%)]	36 (14.5)	26 (19.2%)	13 (11.5)	0.094
Single ASCT after induction [n (%)]				0.340
Yes	157 (67.4%)	81 (65.8)	76 (69.1)	
No	76 (32.6%)	42 (34.1)	34 (30.9)	

### FISH analysis of cytogenetic abnormalities

In the FISH results of 248 patients, 95.9% (238/248) were sorted by CD138, and the rest were not sorted due to the lack of specimens. Among them, 135 (54.4%) cases had 1q21 gain/amplification, 34 (13.7%) cases had 17p deletion, 130 (52.4%) cases had 13q deletion, 55 (22.2%) cases had t(4;14) abnormality, 24 (22.2%) cases had t(4;14) abnormality, 24 (9.7%) cases had t(11;14) abnormality, 2 (0.8%) cases had t(14;16) abnormality, and 32 (12.9%) patients were only positive for 1q21 without any of the above genetic abnormalities. There were 51 (20.6%) of double-hit and 4 (1.6%) of triple-hit, as shown in **Table 2**. Compared with the 1q21 negative group, 1q21+ patients were more likely to accompanied with 13q14 deletion (P=0.001) and t(4;14) (P=0.014), and less likely to combine 17p deletion (P=0.025).

**Table 2:** Cytogenetic abnormalities of patients with total patients and 1q21+ patients detected by FISH.

Cytogenetic abnormalities	Total (n=248)	1q21+ (n=135)	1q21- (n=113)	P value
13q14 deletion	130 (52.4%)	86 (63.7%)	44 (38.9%)	0.001
17p deletion	34 (13.7%)	12 (8.8%)	22 (19.4%)	0.025
t(4;14)	55 (22.2%)	38 (28.1%)	17 (15.0%)	0.014
t(11;14)	24 (9.7%)	11 (8.1%)	13 (11.5%)	0.373
t(14;16)	2 (0.8%)	2 (1.5%)	0 (0%)	0.193
mSMART3.0 double-hit	51 (20.6%)	44 (32.6%)	7 (6.1%)	<0.0001
mSMART3.0 triple-hit	4 (1.6%)	4 (3.0%)	0 (0%)	0.065

**Figure 1:**
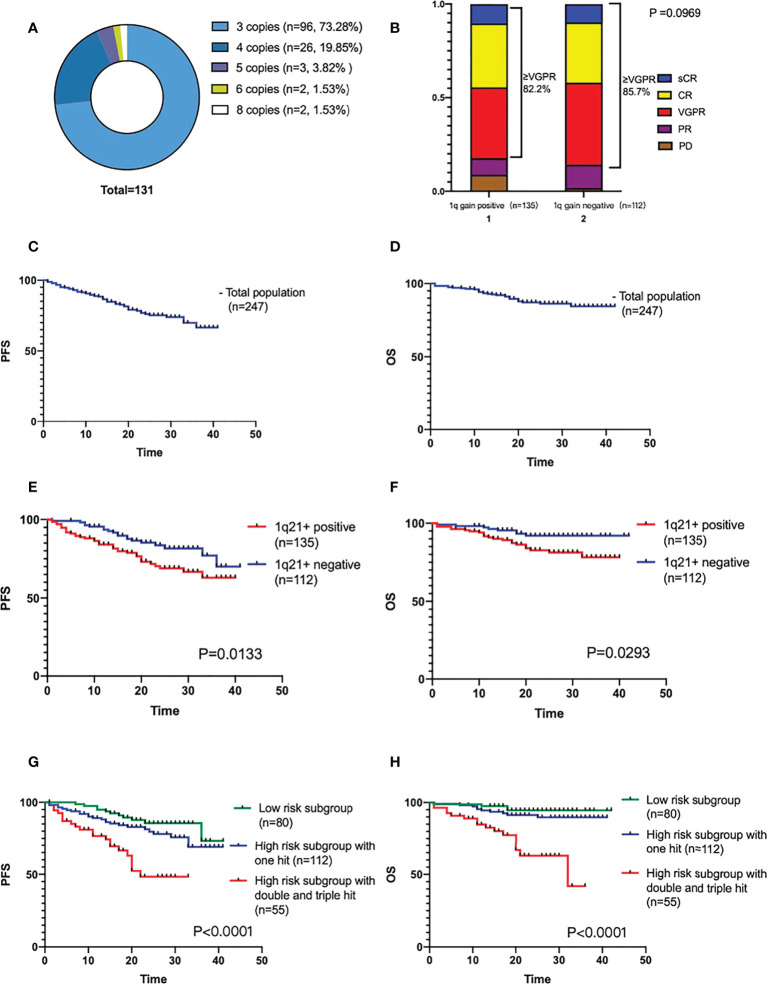
**(A)** Different 1q21+ positive copy numbers detected by FISH. **(B)** Efficacy of induction therapy in patients with or without 1q21+. **(C, D)** The PFS and OS analysis of the total population. **(E, F)** The PFS and OS comparison of 1q21+ positive and negative patients. **(G, H)** The PFS and OS comparison by mSMART3.0 stratified risk stage.

4 of 135 1q21+ patients were reported by other hospitals, and the specific positive ratio and copy numbers were not reported. Among the remaining 131 patients, 24 cases (18.3%, including 8 cases without CD138 sorting) had a positive rate of 0-20%, and 20 cases (15.3%) with a rate of 21-50%, and 89 (67.9%) with a rate of 51-100%. 1q21 gain with 3 copies in 96 (73.3%) patients, and the ≥4 copies in the rest 35 (26.7%) patients, as shown in **Figure 1A**. In this study, a total of 15 (6.1%) patients were “double hit” high-risk patients according to the definition by Walker BA that ISS stage III combined with 1q21 amplification (≥4 copies).

### CytoScan analysis of cytogenetic abnormalities

153 patients’ samples were tested by Cytoscan along with the FISH tests, and a total of 145 (94.8%) patients were detected with abnormal karyotypes. A total of 77 (50.3%) patients were positive for 1q21 gain/amplification with CKS1B involvement, and 3 (2.0%) patients were positive for 1q21 gain/amplification without CKS1B involvement, and 4 patients were positive for 1q gain but not involved in 1q21 locus. Compared with the negative group, the differences in proportion of complex karyotype and CNV abnormality were clinically significant (P<0.0001). Moreover, patients in the positive group had more subclones (P=0.0001) and the chromosomal karyotypes were mainly hypodiploidy (46.8%). The proportion of +5 with good prognosis (P=0.0487) was low in this group, and it was easier to merge with 1p32 deletion (P=0.0002). All of them were in the middle-risk or high-risk group by PI score (P<0.0001), as shown in **Table 3**.

**Table 3:** Chromosomal abnormalities in 1q21+ patients and CKS1B involvement detected by CytoScan.

Chromosomal abnormalities	Total (n=153)	1q21+ (n=77)	1q21- (n=86)	P value
Complex karyotype [n, (%)]	107 (69.9)	63 (81.8)	44 (51.2)	<0.0001
CNV abnormality [n, (%)]	116 (75.8)	71 (92.2)	45 (52.3)	<0.0001
Subclone [n, (%)]				0.0001
0	41 (26.8)	10 (13.0)	31 (36.0)	
1	48 (31.4)	24 (31.2)	24 (27.9)	
2	64 (41.8)	43 (55.8)	21 (24.4)	
Karyotype [n, (%)]				<0.0001
Diploid	49 (32.0)	13 (16.9)	36 (41.9)	
Hypodiploid	53 (34.6)	36 (46.8)	17 (19.8)	
Small hypodiploid	21 (13.7)	16 (20.8)	5 (5.8)	
Hyperdiploid	30 (19.6)	12 (15.6)	18 (20.9)	
With +5 [n, (%)]	27 (17.6)	8 (10.4)	19 (22.1)	0.0487
With +21 [n, (%)]	19 (12.4)	8 (10.4)	11 (12.8)	0.6549
With 1p32- [n, (%)]	18 (11.8)	16 (20.8)	2 (2.3)	0.0002
PI score [n, (%)]				<0.0001
≤0 score (low-risk)	46 (30.1)	0 (0)	46 (53.5)	
0-1 score (middle-risk)	72 (47.1)	52 (67.5)	11 (12.8)	
≥1 score (high-risk)	35 (22.9)	25 (32.5)	19 (22.1)	

### Analysis of the inconsistency of 1q21+ by FISH and CytoScan in 153 cases

The consistence rate of FISH and CytoScan for the detection of 1q gain/amplification was 90.8%. 77 cases were detected by both methods of 1q21+ and CKS1B involvement, and 59 cases were negative by both methods.

Correction of FISH results by CytoScan in 9 patients showed that 2 patients had hyperdiploidy involving trisomy 1 and were FISH positive. 1 patient had 1q gain without involving 1q21 and was FISH positive (positive proportion<20%). 3 patients had 1q gain without involving 1q21 and were FISH negative. 3 patients had 1q21 gain but not with CKS1B involvement and were FISH negative. The other 8 patients were positive by FISH but negative by Cytoscan (3 case with positive proportion<20%).

### Treatment response and survival status

All 248 patients were followed up until February 28, 2022, and one of them was lost follow-up. 247 patients underwent efficacy evaluation after induction therapy were included in the final analysis. 26 cases were PR, 100 cases were VGPR, 82 cases were CR, and 25 cases were sCR. The ORR of the total population was 94.3%, ≥VGPR was 83.8%, and the ≥CR ratio was 43.3%. There was no clinical significance in the treatment response analysis of the two subgroups (P=0.0969), as shown in **Figure 1B**.

The median follow-up period was 20 (1-42) months. Neither median PFS nor OS was reached. The cumulative PFS at 40 months was 65.9%, and the cumulative survival rate at 40 months was 84.4%, as shown in **Figures 1C, D**.

### 1q21+ and R-ISS stage

1q21+ has a significant impact on the prognosis of NDMM patients. The survival analysis of the two patient subgroups (1q21+, n=135; 1q21-, n=112) showed that the PFS and OS were worse in the positive patient group (the median PFS was not reached, P=0.0133; median OS not reached, P=0.0293), as shown in **Figures 1E, F**.

According to the mSMART3.0, they were divided into low-risk group (n=80), high-risk single-hit group (n=112), high-risk double-hit or triple-hit group (double-hit n=51, triple-hit n=4) for survival analysis which showed that the double-hit and triple-hit group had the worst prognosis, with a median PFS of 22 months and a median OS of 32 months. Both PFS and OS were clinically significant (PFS P<0.0001, OS P<0.0001), as shown in **Figures 1G, H**.

### 1q21+ copy numbers and positive proportion

**Figure 2:**
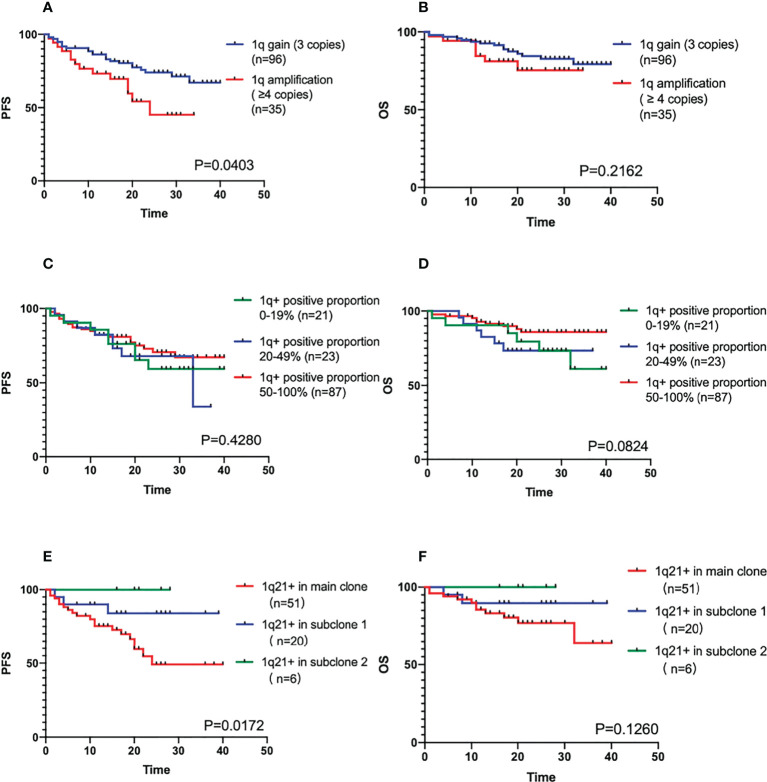
**(A, B)** The PFS and OS comparison of different positive copy numbers in 1q21+ patients. **(C, D)** The PFS and OS comparison of different positive ratio in 1q21+ patients. **(E, F)** The PFS and OS comparison of main clone and subclone in 1q21+ patients detected by Cytoscan.

We also divided 1q21+ patients into 1q21 gain (3 copies) (n=96) group and 1q21 amplification (≥4 copies) (n=35) group for comparison. The results showed that the PFS of 1q21 amplification patient group was shorter (median PFS was 24 months vs. Not reached, P=0.0403), but the difference in OS between the two groups was not clinically significant (the median OS was not reached, P=0.2162) in **Figures 2A, B**. The positive ratio of 1q gain/amplification had no effect on the survival of patients (PFS P=0.4280, OS P=0.0824), as shown in **Figures 2C, D**.

### 1q21+ in main clone and subclone

There were 77 patients positive for 1q21 gain/amplification with CKS1B involvement by CytoScan, and 51 patients’ 1q positive were in the main clone. When the proportion of abnormal clones differed by 30%, it was generally considered that were subclones. There were 20 cases in the subclone group 1 with a larger proportion, and 6 cases in the subclone group 2 with a smaller proportion. According to the results of the survival analysis, whether the gain/amplification in the main clone was an adverse factor affecting the prognosis of the patients (the median PFS was not reached, P=0.0172, the median OS was not reached, P=0.1260), as shown in **Figures 2E, F**.

### ASCT in 1q21+ patients

**Figure 3:**
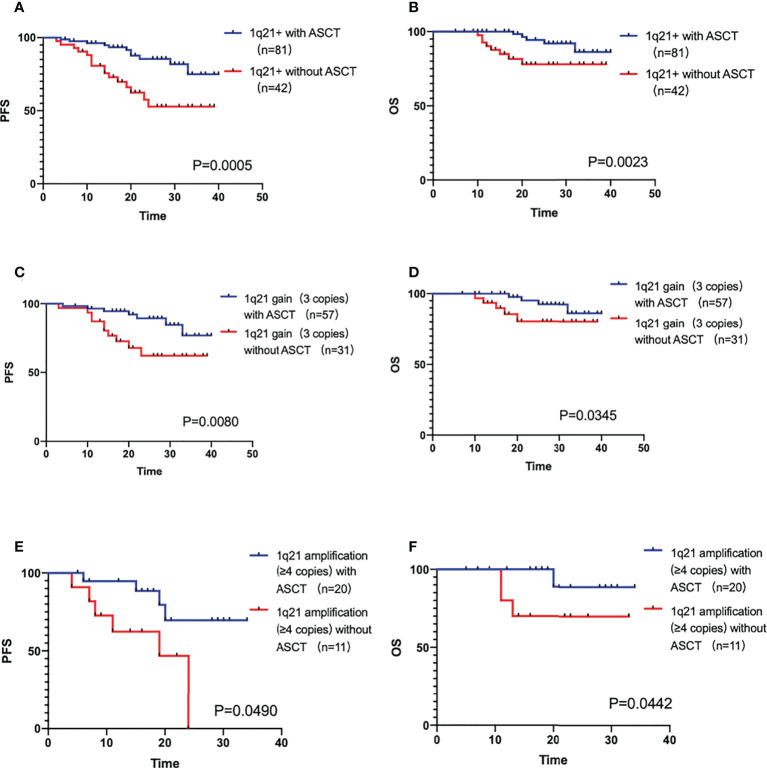
**(A, B)** The PFS and OS comparison of 1q21+ patients with ASCT and without ASCT. **(C, D)** The PFS and OS comparison of 1q21 gain patients with ASCT and without ASCT. **(E, F)** The PFS and OS comparison of 1q21 amplification patients with ASCT and without ASCT.

In the 135 1q21+ patients, 12 of them disease progressed in induction treatment period, the rest 123 patients had completed pre-transplantation therapy. According to whether ASCT was performed or not, the patients were divided into a transplantation group (n=81) and a non-transplantation group (n=42). The median PFS and OS were not reached in both groups. ASCT could significantly improve the prognosis of patients (PFS P=0.0005, OS P=0.0023). In the 1q gain sub-population, transplantation group (n=57) compared with non-transplantation (n=31) group showed a clinically meaningful improvement in prognosis (PFS P=0.0080, OS P=0.0345). Results were similar in the 1q amplification sub-population (PFS P=0.0490, OS P=0.0442), with a median PFS of 19 months in the non-transplantation group (n=11) and not reached in the transplantation group (n=20), as shown in **Figures 3A–F**).

### Univariate analysis of the prognosis of multiple myeloma

Univariate analysis of varies clinical and cytological factors was performed on 247 NDMM patients, including age, gender, M protein type, R-ISS stage, hemoglobin, total protein, serum Ca^2+^, serum creatinine, treatment response, single transplantation and different cytogenetic abnormalities. The results of univariate analysis showed that NDMM patients with age ≥65 years old, R-ISS stage III, HGB ≤ 100g/L, creatinine≥177μmol/L, induction therapy efficacy≤CR, without single transplantation or combined with 1q21+, 17p deletion or t(4; 14) had significant inferior outcomes in PFS (P<0.05). As same in OS results, NDMM patients with age ≥65 years old, R-ISS stage III, HGB ≤ 100g/L, creatinine ≥177μmol/L, Ca^2+^≥2.65 mmol/L, induction therapy efficacy ≤CR, without single transplantation or combined with 1q21+ or t(4; 14) had inferior outcomes in OS (P<0.05), as shown in **Attachment Table 1**. There were 135 patients with 1q21+, and there are 78 patients without any of 17p-\t(4;14)\t(11;14)\t(14,16) factors. The results show the singke 1q21+ population co-segregating with any of these factors still has a poor prognosis of PFS and OS significantly (PFS HR=0.2624, 95%CI 1.363-5.052, P=0.004, HR=4.036, 95%CI 1.625-10.021, P=0.003).

### Multivariate analysis of the prognosis of multiple myeloma

**Figure 4:**
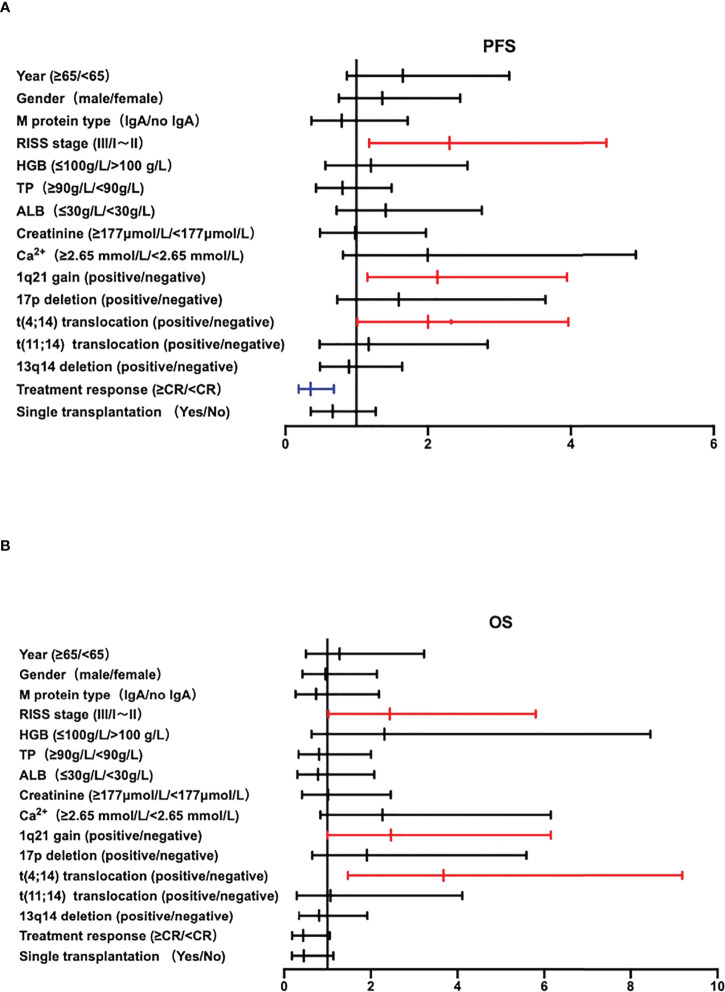
**(A, B)** The Forest plot of PFS and OS of multivariate analysis of prognostic factors in NDMM patients.

Multivariate analysis was performed on 248 NDMM patients, combined with the results of univariate analysis that clinical and cytological characteristics such as age, gender, M protein type, R-ISS stage, hemoglobin, total protein, serum Ca^2+^, serum creatinine, treatment response, single transplantation and different cytogenetic abnormalities. R-ISS, 1q21+, translocation 11,14 and CR were independent adverse factors associated with poor prognosis of PFS, and R-ISS, 1q21+, translocation 11,14 is important for OS. Especially 1q21+ was an independent adverse factor associated with poor prognosis of NDMM patients (PFS: HR=2.133, 95%CI 1.153-3.945, P=0.016; OS: HR=2.246, 95%CI 1.002-6.067, P=0.049), as shown in **Attachment Table 2** and **Figures 4A, B**.

## Discussion

Research of 1q21+ in risk stratification were vary globally. In the Total Therapy 2 trial (14), 1q21+ was first reported as an adverse cytogenetic aberration in MM. The 2014 IMWG consensus classified 1q21+ as a standard-risk abnormality, while low-risk must be consistent with negative 1q21+ (15). mSMART3.0 (2018) defined 1q21+ as a high-risk abnormality for the first time and defined”very high-risk MM”subgroups such as double-hit and triple-hit (16). Dr Walker defined “double-hit high-risk “ when 1q21 amplification (≥4 copies) appears with ISS stage III, or simultaneous has biallelic inactivation of TP53 (17). Combined with the PI score detected by CytoScan (18), the prognostic score was obtained according to different additional coefficients, and 1q21 was a poor prognostic factor. However, diagnosis and treatment guidelines in China uses R-ISS to define high-risk which is FISH detected del(17p), t (4;14), and t (14;16). The prognostic significance of 1q21+ in NDMM patients has not been clarified in the guideline which may be influenced by the susceptibility of 1q to coexist with additional chromosomal aberrations and/or concomitant other biological risk factors.

Among the 248 NDMM patients in this study, 54.4% patients had 1q21+, which was similar to 59.7% (96/196) positive detection rate of 1q21+ by FISH in the study by Gao et al. in 2022 (4). By way of illustration, Mao (5) shows the data of a MM cytogenetics retrospective study (47.4%, 376/789) (7), and which is also higher than that reported in Western countries. According to a statistical analysis (7) with a meta-analysis of 2,596 trial patients from GMMG (HD4 and MM5 combined) and MyXI trial patients, with 1q21 abnormalities in 37% (324/880) and 34% (577/1716), and the P values are both <0.0001, which confirms that 1q21 gain/amplification is higher in the Chinese population 54.4%(135/248). It is closely related to the situation that MM patients may have long disease duration and late stage when initial diagnosed in China. Patients with 1q21+ are more likely to develop end-stage organ damage, higher tumor burden, and are more likely to be combined with 13q14 deletion, t(4;14), and 1p deletion. In addition, those with CKS1B involvement are more likely to have a high proportion of complex karyotypes, large fragmental CNV abnormalities, with more subclones, belong to the PI-defined high-risk group.

Univariate and multivariate analysis indicated that 1q21+ with the near value of HR like 17pdeletion and t(4;14), was an independent high-risk cytogenetic factor for poor prognosis in NDMM. Further analysis of the biological properties of 1q21+ is required. Firstly, the impact of 1q21+ copy number on the prognosis of MM needs more attention. Neben et al. reported (19) that both 1q21 gain and amplification were associated with reduced PFS and OS, and the effect was more significant in patients with 1q21 amplification. In a study of 286 cases with NDMM, An et al (20) demonstrate that copy numbers of 1q21 increased with progression of myeloma, but Yu et al. (21) study did not find the significant difference of OS between patients with 1q21 gain or amplification. The results of our study suggested that 1q21 amplification had worse PFS than 1q21 gain (24 months vs not reached, P=0.0403). The next is the 1q21 positive proportion, a recent study showed that patients with a 1q21+ clone size of 5%-20% had a significantly lower 2-year PFS rate than 1q21- patients (P=0.041), and no significant difference compared with that of patient clone size >20% on PFS rate (4). The data of our study showed that the positive ratio of 1q21 had no effect on the survival of patients (PFS P=0.4280, OS P=0.0824). Moreover, the CytoScan test results showed that 1q21+ located in the main clone had a worse prognosis than that of the subclone subgroup (P=0.0172). Therefore, it will be essential for clinicians to evaluate for and document the presence or absence of an abnormality, especially the copy number and clone status of 1q21 in all patients, and to report this data in a uniform matter, alongside other frequently reported cytogenetics such as IgH translocations and del(17p).

ASCT plays an important role in the prognostic survival of 1q21+ patients, both 1q gain and 1q amplification, P value of PFS and OS all <0.05. Not only that, past studies studied the CRD/CTD scheme, KRd/KCD scheme or VRD are in a subgroup control (22), our study focus on the patients with VRD +/- ASCT from a registration study is also unique, demonstrating that the prognosis of 1q21 in VRD scheme is also poor, but ASCT can overcome the adverse effects on the patients significantly.

Since chromosome cytogenetic studies and their clinical effects analysis of multiple myeloma have achieve great success and tremendous progress. Our center mainly intends to explore more of the complex whole gene sequencing of MM to establish the gene profile of multiple myeloma in Chinese population. Taking chromosome 1 for example, the University of Arkansas Research Institute found 70 gene markers associated with early progression, 30% of which were mapped to chromosome 1 (23). Most of the up-regulated genes are located on chromosome 1q, such as CKS1B, PSMD4, IL6R, ADAR, MCL1, etc. in tumor proliferation, among which CKS1B acts as gene encoding a cofactor for the Skp2-dependent ubiquitination of p27^kip1^ (24), which negatively regulates the G1/S transition of the cell cycle, thereby promoting cell proliferation and leading to disease progression (25). The down-regulated genes are located on chromosome 1p, such as 1p12 (FAM46C), 1p22.1 (RPL5) and 1p32.3 (CDKN2C). CDKN2C acts as a tumor suppressor gene, and its deletion will also affect cell cycle regulation (26). The phenomenon may explains the intrinsic mechanism of 1q and 1p in clinical prognosis, so it’s worthy of more clinical and laboratory work for complex abnormalities.

CytoScan technology can analyzes DNA sequence polymorphisms caused by variation at the nucleotide level of the genome, by now analysis and interpretation of whole genome copy number variations (CNVs) is quite a challenging task for clinicians. So it hasn’t been widely applied for clinical use in China, however our study has proved the clinical practicability and effectiveness of this technology, which is worth popularizing. The prevalence of 1p32 deletion in this study was 11.8%, which is consistent with the reported rate of 7.3%-15% (26), and the data showed that 1q21 positive patients were more likely to have 1p32 deletion (P=0.0002). The previous guidelines required that the 1p32 locus was not included in the FISH test. Hebraud verified 1p32 as an independent poor prognostic factor for MM through IFM1195 patients (27), by using CytoScan technology, the 1p high-risk deletion and related involved gene can be accurately detected.

By combining FISH, CytoScan and other technologies with focusing on 1q21 and other related abnormalities such as del(17p13), t (4;14), t(14;16), 1p32 deletion, etc., clinical characteristics, efficacy evaluation, and prognosis could be analyzed more accurately. Although there is no global consensus on risk stratification, R-ISS, IMWG, mSMART, Prognostic Index score and other stratification systems using different biological indicators are all aimed at identifying patients might have poor prognosis. A more comprehensive genomic detection method could be more accurately classify the disease prognosis. At the same time, it can also analyze the differences of different drugs in different myeloma risk groups, further enhancing the possibility of moving forward to the treatment strategy based on risk stratification in the future.

There are still deficiencies in this study, regarding the genetic abnormalities represented by the 1q21 related to multiple myeloma, it is still necessary to expand the sample size, include multi-center clinical data, and prolong the follow-up, as well as continue to promote comprehensive implementation of FISH, CytoScan and other technologies in MM patients to further understand the impact of cytogenetic and clinical characteristics on the prognosis of high-risk multiple myeloma patients.

## Data availability statement

The original contributions presented in the study are included in the article/**Supplementary Material**. Further inquiries can be directed to the corresponding authors.

## Ethics statement

The studies involving human participants were reviewed and approved by the ethics committee of the First Affiliated Hospital of Soochow University. The patients/participants provided their written informed consent to participate in this study.

## Author contributions

HY, SJ, and CW designed the study, collected the data, analyzed the data, and wrote the article. QW, SY, WY, XS, JS, and LY contributed to data collection and analysis. YY, JW, PW, and DW contributed to the data collection and verification. JP and CF contributed to the research design, data analysis, article composition, and study supervision. All authors contributed to the article and approved the submitted version.

## Funding

This study was supported by Translational Research Grant of NCRCH (Grant No. 2020ZKPB01), Suzhou Project of Science and Technology 2021 (Grant No. SLJ2021004), and the China Primary Health Care Foundation.

## Acknowledgments

We wish to thank Feiran Gong, Jiazi Zhou, Li Yao, Hongjie Shen and Hong Liu for assistance in data collection and verification.

## Conflict of interest

The authors declare that the research was conducted in the absence of any commercial or financial relationships that could be construed as a potential conflict of interest.

## Publisher’s note

All claims expressed in this article are solely those of the authors and do not necessarily represent those of their affiliated organizations, or those of the publisher, the editors and the reviewers. Any product that may be evaluated in this article, or claim that may be made by its manufacturer, is not guaranteed or endorsed by the publisher.
