# Supplementary material for: The independent adverse prognostic significance of 1q21 gain/amplification in newly diagnosed multiple myeloma patients
Source: Front Oncol. 2022 Oct 7;12:938392. doi: 10.3389/fonc.2022.938392 (PMC9585234; doi:10.3389/fonc.2022.938392)
Supplement: Supplementary file 1 [file DataSheet_1.docx]

**Attachment Table 1. Univariate analysis of prognostic factors in NDMM patients**

| Univariate analysis | PFS | | | | OS | | | |
| --- | --- | --- | --- | --- | --- | --- | --- | --- |
|  | P value | HR | 95% CI | | P value | HR | 95% CI | |
| Year (≥65/<65） | <0.0001 | 2.628 | 1.548 | 4.462 | 0.020 | 2.370 | 1.148 | 4.894 |
| Gender（male/female） | 0.057 | 1.699 | 0.984 | 2.932 | 0.501 | 1.285 | 0.619 | 2.670 |
| M protein type（IgA/no IgA） | 0.634 | 0.847 | 0.428 | 1.677 | 0.614 | 0.781 | 0.299 | 2.041 |
| RISS stage (III/I～II） | <0.0001 | 3.581 | 2.094 | 6.126 | <0.0001 | 5.215 | 2.542 | 10.701 |
| HGB (≤100g/L/>100 g/L） | 0.035 | 2.030 | 1.051 | 3.920 | 0.020 | 4.103 | 1.245 | 13.528 |
| TP（≥90g/L/<90g/L) | 0.946 | 0.982 | 0.578 | 1.667 | 0.694 | 0.861 | 0.410 | 1.810 |
| ALB（≤30g/L/<30g/L) | 0.100 | 0.643 | 0.380 | 1.089 | 0.651 | 1.187 | 0.565 | 2.496 |
| Creatinine（≥177μmol/L/<177μmol/L） | 0.025 | 1.911 | 1.083 | 3.371 | 0.016 | 2.499 | 1.189 | 5.252 |
| Ca2+（≥2.65 mmol/L/<2.65 mmol/L) | 0.155 | 1.782 | 0.804 | 3.950 | 0.023 | 2.826 | 1.153 | 6.927 |
| 1q21 gain (positive/negative) | 0.023 | 1.898 | 1.094 | 3.293 | 0.025 | 2.521 | 1.122 | 5.665 |
| 17p deletion (positive/negative) | 0.022 | 2.111 | 1.115 | 3.996 | 0.053 | 2.307 | 0.989 | 5.384 |
| t(4;14) translocation (positive/negative) | 0.006 | 2.193 | 1.247 | 3.857 | 0.001 | 3.399 | 1.644 | 7.029 |
| t(11;14) translocation (positive/negative) | 0.339 | 1.472 | 0.666 | 3.252 | 0.872 | 1.103 | 0.334 | 3.641 |
| 13q14 deletion (positive/negative) | 0.256 | 1.357 | 0.802 | 2.296 | 0.163 | 1.698 | 0.808 | 3.569 |
| Treatment response (≥CR/<CR） | 0.001 | 0.362 | 0.198 | 0.662 | 0.045 | 0.438 | 0.195 | 0.983 |
| Single transplantation (Yes/No) | <0.0001 | 0.363 | 0.215 | 0.613 | 0.002 | 0.306 | 0.147 | 0.636 |
| Single 1q21+ (Yes/No) (n=153) | 0.004 | 2.624 | 1.363 | 5.052 | 0.003 | 4.036 | 1.625 | 10.021 |

**Attachment Table 2. Multivariate analysis of prognostic factors in NDMM patients**

| Prognostic factors | PFS | | | | OS | | | |
| --- | --- | --- | --- | --- | --- | --- | --- | --- |
|  | P value | HR | 95% CI | | P value | HR | 95% CI | |
| Year (≥65/<65） | 0.128 | 1.648 | 0.865 | 3.138 | 0.601 | 1.280 | 0.507 | 3.231 |
| Gender (male/female) | 0.305 | 1.361 | 0.755 | 2.451 | 0.915 | 0.957 | 0.427 | 2.144 |
| M protein type (IgA/no IgA) | 0.559 | 0.795 | 0.368 | 1.717 | 0.592 | 0.744 | 0.253 | 2.191 |
| RISS stage (III/I～II） | 0.015 | 2.303 | 1.178 | 4.503 | 0.044 | 2.441 | 1.026 | 5.807 |
| HGB (≤100g/L/>100 g/L) | 0.632 | 1.202 | 0.566 | 2.553 | 0.204 | 2.316 | 0.634 | 8.456 |
| TP (≥90g/L/<90g/L) | 0.490 | 0.805 | 0.434 | 1.492 | 0.648 | 0.810 | 0.327 | 2.005 |
| ALB (≤30g/L/<30g/L) | 0.314 | 1.410 | 0.722 | 2.754 | 0.628 | 0.786 | 0.297 | 2.082 |
| Creatinine (≥177μmol/L/<177μmol/L) | 0.962 | 0.983 | 0.490 | 1.972 | 0.976 | 1.014 | 0.417 | 2.464 |
| Ca2+ (≥2.65 mmol/L/<2.65 mmol/L) | 0.132 | 1.997 | 0.812 | 4.913 | 0.107 | 2.271 | 0.839 | 6.152 |
| 1q21 gain (positive/negative) | 0.016 | 2.133 | 1.153 | 3.945 | 0.049 | 2.466 | 1.002 | 6.067 |
| 17p deletion (positive/negative) | 0.243 | 1.591 | 0.730 | 3.465 | 0.237 | 1.910 | 0.653 | 5.590 |
| t(4;14) translocation (positive/negative) | 0.047 | 2.002 | 1.011 | 3.964 | 0.005 | 3.680 | 1.473 | 9.190 |
| t(11;14) translocation (positive/negative) | 0.728 | 1.170 | 0.483 | 2.834 | 0.917 | 1.074 | 0.280 | 4.114 |
| 13q14 deletion (positive/negative) | 0.722 | 0.896 | 0.490 | 1.640 | 0.632 | 0.810 | 0.341 | 1.921 |
| Treatment response (≥CR/<CR） | 0.002 | 0.358 | 0.187 | 0.685 | 0.065 | 0.438 | 0.183 | 1.051 |
| Single transplantation （Yes/No) | 0.225 | 0.677 | 0.360 | 1.271 | 0.091 | 0.453 | 0.181 | 1.135 |
